# Day-to-day variations during clinical drug monitoring of morphine, morphine-3-glucuronide and morphine-6-glucuronide serum concentrations in cancer patients. A prospective observational study

**DOI:** 10.1186/1472-6904-4-7

**Published:** 2004-10-04

**Authors:** Pål Klepstad, Priscilla Hilton, Jorunn Moen, Stein Kaasa, Petter C Borchgrevink, Kolbjørn Zahlsen, Ola Dale

**Affiliations:** 1 Department of Circulation and Imaging, Faculty of Medicine, Norwegian University of Science and Technology, N-7489 Trondheim, Norway; 2 Unit for Applied Clinical Research, Department of Cancer Research and Molecular Medicine, Norwegian University of Science and Technology, Trondheim, Norway; 3 Department of Clinical Pharmacology, St. Olavs University Hospital, Trondheim, Norway

## Abstract

**Background:**

The feasibility of drug monitoring of serum concentrations of morphine, morphine-6-glucuronide (M6G) and morphine-3-glucuronide (M3G) during chronic morphine therapy is not established. One important factor relevant to drug monitoring is to what extent morphine, M6G and M3G serum concentrations fluctuate during stable morphine treatment.

**Methods:**

We included twenty-nine patients admitted to a palliative care unit receiving oral morphine (n = 19) or continuous subcutaneous (sc) morphine infusions (n = 10). Serum concentrations of morphine, M6G and M3G were obtained at the same time on four consecutive days. If readmitted, the patients were followed for another trial period. Day-to-day variations in serum concentrations and ratios were determined by estimating the percent coefficient of variation (CV = (mean/SD) ×100).

**Results:**

The patients' median morphine doses were 90 (range; 20–1460) mg/24 h and 135 (range; 30–440) mg/24 h during oral and sc administration, respectively. Intraindividual fluctuations of serum concentrations estimated by median coefficients of day-to-day variation were in the oral group for morphine 46%, for M6G 25% and for M3G 18%. The median coefficients of variation were lower in patients receiving continuous sc morphine infusions (morphine 10%, M6G 13%, M3G 9%).

**Conclusion:**

These findings indicate that serum concentrations of morphine and morphine metabolites fluctuate. The fluctuations found in our study are not explained by changes in morphine doses, administration of other drugs or by time for collection of blood samples. As expected the day-to-day variation was lower in patients receiving continuous sc morphine infusions compared with patients receiving oral morphine.

## Background

Morphine is degraded in the liver to several metabolites of which morphine-6-glucuronide (M6G) and morphine-3-glucuronide (M3G) are biological active [1]. M6G is shown to contribute to the analgesia produced by morphine and may cause opioid related adverse effects such as sedation or nausea [2-5]. Due to first pass metabolism and slow accumulation of M6G in the brain the analgesic activity of M6G is most prominent during oral long-term treatment with morphine while single dose studies show less contribution from M6G to the analgesic effects from morphine [2,3,6]. M3G may in exceptional cases cause excitatory adverse effects such as delirium, myoclonus or allodynia [7]. Animal studies observed that M3G have an anti-nociceptive effect [8,9], but this effect was not reproduced in a study administering M3G to volunteers exposed for human experimental pain [10].

The most obvious determinants for serum concentrations of morphine, M6G and M3G are morphine doses, route of administration and renal function. However, a considerable variation of serum concentrations between patients remains after correcting for dose and route of administration [3,11-13].

Measurements of morphine, M3G and M6G serum concentrations can explain individual responses in patients where morphine treatment turns out to have unexpected effects and help physicians to determine changes in pain treatment. Physicians tend to believe that samples obtained for therapeutic drug monitoring during steady state conditions will be representative irrespective of which day the sample is collected. However, morphine, M6G and M3G serum concentrations may also have fluctuations not caused by changes in morphine doses, administration of other drugs or by time for collection of blood samples. This variation represents the day-to-day variability. In order to evaluate the clinical implications from morphine and metabolites serum concentrations measurements it is necessary to know if these serum concentrations have fluctuations not related to changes in drug administrations.

The day-to-day variability in serum concentrations of morphine, M6G and M3G are previously reported in a study of 8 cancer patients treated with subcutaneous (sc) morphine infusions. This study observed coefficients of variation (CV) ranging from 26%–56% for morphine, 20% to 51% for M6G and 20%–49% for M3G [12]. To our knowledge, the day-to-day variations of morphine, M6G and M3G serum concentrations obtained from consecutive days or during chronic oral administration of morphine are not previously reported.

Thus, the aim of this study is to investigate the day-to-day variations of morphine, M6G and M3G serum concentrations during stable chronic oral and sc morphine administration to cancer patients.

## Methods

### Patients

We included twenty-nine patients admitted during a nine-month period to the Palliative Care Unit at the University Hospital in Trondheim. The inclusion criteria were; verified malignant disease, expected survival time less than 6 months, scheduled morphine treatment started at least three days prior to inclusion, stable scheduled doses of morphine for a minimum of three days and age more than eighteen years. The exclusion criteria were; planned hospitalisation less than three days and lack of ability to communicate (e.g. dementia, deafness).

All patients gave their written informed consent before inclusion. The study was conducted according to the guidelines of the Helsinki declaration. The Regional Committee for Medical Research Ethics, Health Region IV, Norway, approved the study.

### Study design

#### Inclusion

The patients were included in the study within three days after admission to the Palliative Care Unit. Each patient was followed for four days. Patients readmitted to the Palliative Care Unit were allowed to a new trial period identical to the first trial period. No patients were included in more than three trial periods.

The patients' age, gender, primary malignant diagnosis, presence of metastasis, and other medications were registered. Morphine treatment during the last 24 hours was registered with respect to route of administration, morphine formulation, scheduled dose and consumption of rescue morphine for breakthrough pain. The patients' functional status was assessed using the Karnofsky performance status score [15].

#### Blood samples

Blood samples were obtained each day during the trial period. The samples were obtained at the same time each day during the routine morning round for collecting blood samples.

#### Observations

In order to observe if the patients were studied during stable treatment conditions the scheduled morphine dose, rescue morphine consumption and route of administration were registered each study day. The use of other medications was also registered daily. Pain, nausea and sedation were assessed at day study two, three and four during the trial period using a 5 category verbal rating scale (VRS) score ranging from no to very severe. All symptoms were assessed for the last 24 h.

#### Analyses

The blood samples were placed in EDTA tubes until separated by centrifugation (3000 rpm, ten minutes) and stored at -85°C until analysed. All samples were analysed for serum concentrations of morphine, M6G and M3G applying liquid chromatography mass spectrometry [16]. The limits of detection were for morphine 0,35 nmol/l and for M6G and M3G 2,2 nmol/l. The analytical coefficients of variation obtained in quality control samples (CV_Analytical_) were for morphine 3,0%, for M6G 5,5% and for M3G 7,0%. The analytical coefficients of variation were determined at 100 nmol/l for morphine and 1000 nmol/l for M6G and M3G. Serum values of creatinine concentrations, alanin aminotransferase activities (ALAT), aspartat aminotransferase activities (ASAT) and albumin concentrations were determined using standard analytical methods.

#### Statistical evaluation

Total use of morphine for each trial day was calculated by adding scheduled morphine doses and rescue morphine consumption. Samples obtained less than two hour after the administration of a morphine rescue dose were excluded from the analyses.

Day-to-day variations of morphine and its metabolites are presented as biological coeffecients of variation. This biological variation (CV_Biological_), expressed in terms of percent coefficient of variation, was calculated for each patient in each trial period using the equation [15,16]:

*CV*_*Biological *_= *CV*_*Observed *_- *CV*_*Analytical*_

The observed coefficients of variation (CV_Observed_) for morphine, M6G and M3G, which represent the variation in serum concentrations for each patient during each trial period, were calculated using the equation [17,18]:



At least three observations were needed in order to calculate an observed coefficient of variation.

Statistical comparisons between the trial days and trial periods were performed using one-way analysis of variance tests. Due to multiple comparisons statistical significance was defined as p < 0,01.

The statistical software SPSS version 9.0 for Windows was used throughout the analyses.

## Results

### Patient characteristics

The patients (16 males and 13 women) median age at inclusion was 68 years (range; 39–89). The patients' Karnofsky performance status, primary tumor diagnoses and presence of metastases are shown in Table 1. The median serum creatinine concentrations at inclusion were 72 μmol/l (range; 45–121). The median values at inclusion of ASAT and ALAT were 31 IU/l (range; 7–154) and 17 IU/l (range; 5–65), respectively. No patient had clinical significant liver failure. The median serum albumin concentrations at inclusion were 32 g/l (range; 23–42).

**Table 1:** Patient characteristics

Karnofsky performance status (median (range))	60 (40–80)
Cancer Diagnoses	
Prostate	11
Colorectal	5
Kidney	3
Breast	3
Pancreatic	2
Lung	2
Gastric	1
Malignant melanoma	1
Leiomyosarcoma	1
Metastases	
Liver	7
Bone	16
Other	16
Antidepressants	4
Neuroleptics	1
Benzodiazepines	6
Corticosteroids	13
Antiemetics	7

Ten patients used non-opioid analgesics (nine paracetamol, one acetylsalicylic acid). The patients used a median number of 5 (range; 1–10) non-pain medications. The numbers of patients using psychotropic drugs, antiemetics or corticosteroids are given in Table 1. All except one patients received laxatives, lactulose and bisacodyl, during the study period. All medications were stable during the study period. Similar pain, nausea and sedation scores were observed throughout the trial periods (Table 2). Twenty-seven patients had died at the time of manuscript preparation. The median survival time from inclusion was three months.

**Table 2:** Symptom scores All scores were obtained using a 5 category verbal rating scale score (scores; 1–5). All results are given as mean (SD). No significant differences in scores were observed between trial days.

	Trial day 2	Trial day 3	Trial day 4
Pain	2.8 (0.7)	2.2 (1.1)	2.2 (1,1)
Nausea	1.7 (1.0)	1.7 (1.1)	1.8 (0.9)
Sedation	3.4 (1.2)	3.4 (1.0)	3.2 (1.1)

Of the nineteen patients receiving oral morphine sixteen patients completed one trial period, two patients completed two trial periods and one patient completed three trial periods. The corresponding numbers for the ten patients receiving sc morphine were four, five and one, respectively. Six patients were excluded during a study period. The reasons were; discharge from hospital (n = 2), opioid treatment changed to fentanyl patch (n = 1) and fatigue (n = 3). Opioid induced adverse effects caused no exclusions. Sixteen blood samples were not obtained due to circumstances related to the patients' or relatives' needs (e.g. visits from relatives at the time of a planned blood sample).

### Morphine treatment

The median duration of morphine treatment before entering the study was 7 months (range; 0–29). The median morphine dose at inclusion for the patients receiving oral treatment (controlled-release morphine) was 90 mg/24 h (range; 20–1460). The median morphine dose for the patients receiving sc morphine infusions was 135 mg/24 h (range; 30–340). The morphine doses varied between the study days because the patients were allowed to use rescue morphine. This variation, however, was minor (Table 3 and 4).

**Table 3:** Serum concentrations for morphine and metabolites for the oral route The morphine doses (mg/24 h) vary because of variable doses of rescue morphine. A total of 23 trial periods in 19 patients were studied. All data are given as median and range.

	Day 1	Day 2	Day 3	Day 4
Morphine dose (mg/24 h)	90 (20–1460)	80 (20 – 1700)	95 (30 – 1520)	90 (20 – 1580)
Serum morphine (nmol/l)	255 (46–2520)	59 (17–1437)	94 (12–1429)	77 (9–2296)
Serum M6G (nmol/l)	1156 (149–7874)	568 (66–7874)	516 (66–9678)	620 (80–8026)
Serum M3G (nmol/l)	6341 (1734–31997)	3696 (404–36887)	3226 (595–41452)	3778 (526–43043)

**Table 4:** Serum concentrations for morphine and metabolites for the subcutaneous route The morphine doses (mg/24 h) vary because of variable doses of rescue morphine. A total of 17 trial periods in 10 patients were studied. All data are given as median and range.

	Day 1	Day 2	Day 3	Day 4
Morphine dose (mg/24 h)	135 (30–340)	163 (30 – 335)	164 (50 – 440)	150 (84 – 440)
Serum morphine (nmol/l)	240 (42–741)	254 (62–1297)	305 (106–1045)	373 (103–1222)
Serum M6G (nmol/l)	723 (78–1811)	674 (88–2867)	1009 (374–2023)	1225 (400–2339)
Serum M3G (nmol/l)	5350 (578–11784)	4490 (779–16312)	5631 (3028–8342)	6119 (2777–13715)

The diurnal distributions of rescue morphine administrations were recorded in order to assess the possible influence from rescue morphine on the serum concentration observations. Three blood samples were obtained during the two-hour interval following an administration of rescue morphine. The results from these samples were excluded from the analyses.

### Morphine, M6G and M3G serum concentrations

The median serum concentrations of morphine during oral morphine treatment ranged from 59 to 255 nmol/l during the four study days. The median serum concentrations for M6G and M3G on each study day for patients receiving oral morphine are given in Table 3.

The median serum concentrations were more stable during sc morphine treatment compared with oral treatment. The median serum concentrations of morphine during sc morphine treatment ranged from 240 to 373 nmol/l during the study days. The median serum concentrations for M6G and M3G on each study day for patients receiving sc morphine are given in Table 4.

### Day-to-day variation

The median biological coefficient of variation (CV) for morphine serum concentrations was 46% (range; 13–103) during oral morphine therapy and 10% (range; 0–36) during sc morphine infusions (Table 5). The median biological CV values for M6G were 25% (range; 1–72) during oral therapy and 13% (range; 2–40) during s.c. therapy. The corresponding results for M3G serum concentrations were 18% (range; 0–57) and 9% (range; 0–34), respectively (Table 5).

**Table 5:** Biological coefficients of variation (CV) of morphine, morphine-6-glucuronide (M6G) and morphine-3-glucuronide (M3G) serum concentrations during four consecutive days of oral or subcutaneous morphine treatment. All values are given as median and range.

	Biological coefficient of variation %	
	
	Oral morphine	Subcutaneous morphine
Morphine	46 (13–103)	10 (0–36)
M6G	25 (1–72)	13 (2–40)
M3G	18 (0–57)	9 (0–34)

## Discussion

Intraindividual fluctuation of drug serum concentrations not explained by changes in doses, administration of other drugs or by time for collection of blood samples, is the day-to-day variation. Routine measurements of serum concentrations of morphine and metabolites are of questionable value because of the large variability of minimum effective serum concentration and the lack of a direct relationship between serum concentrations and adverse effects [19]. However, measurements of serum concentrations of morphine and metabolites are of importance in patients displaying unexpected opioid toxity [4,7] Physicians assessing results from serum drug concentrations determinations should be aware to what extent serum concentrations of drugs fluctuate during stable treatment conditions. Without this knowledge differences and changes in serum concentrations observations may be unduly interpreted.

The available data on day-to-day variability during chronic morphine treatment is sparse. Vermeire *et al. *reported day-to-day variations during morphine treatment in eight cancer patients receiving continuous sc morphine infusion for 1 to 23 weeks. The individual CV values observed in their study varied between 26% to 56% for morphine, 20% to 51% for M6G and 20% to 49% for M3G [14].

We observed less day-to-day variations of morphine and metabolites concentrations during sc morphine treatment (morphine 10%, M6G 13%, M3G 9%) compared to the fluctuations reported by Vermeire *et al.*. One explanation for this discrepancy is that Vermeire *et al. *obtained blood samples during treatment periods up to 23 weeks. This study design may overestimate day-to-day variability since patient related factors will vary more during long time intervals than between consecutive days.

To our knowledge this is the first study to assess the day-to-day variation of morphine and morphine metabolites serum concentrations during oral morphine therapy. The median observed CV values for serum concentrations of morphine, M6G and M3G during oral morphine therapy (morphine 46%, M6G 25%, M3G 18%) were higher than the CV values observed in patients receiving sc morphine treatment (morphine 10%, M6G 13%, M3G 9%). This observation was expected due to a more stable delivery rate and since absorption during sc administration is not influenced by food intake, gastric retention, malabsorption, vomiting or variable first-pass metabolism.

The results in our study, as in the study by Vermeire *et al.*, represent day-to-day variability in cancer patients admitted to a palliative care unit. In this patient population pharmacological observations will be influenced by variations in food intake, gastric retention, malabsorption, effects from other drugs on gastric emptying, vomiting and drug interactions. In order to perform a study on day-to-day variation not suspect to these confounding factors patients or volunteers must be recruited into a controlled experimental environment. We believe that studies in controlled experimental environments and studies in patients with advanced cancer disease are complementary to each other. The first targets the pharmacokinetic phenomenon of day-to-day fluctuations, the second targets the fluctuations met during clinical real-life conditions.

We recognise some limitations in our study. First, blood samples were collected during four trial days. An extended trial period in order to obtain a larger number of samples from each patient gives a more precise estimate of day-to-day variation. However, due to ethical considerations, taking into account the strain on each patient from serial blood sampling, we chose to not extend the trial periods beyond four days. A second potential confounding factor is absorption peaks in serum concentrations caused by rescue doses of morphine. We chose to allow for rescue morphine because we wanted to observe the variability of serum concentrations as observed in a normal clinical setting in patients considered to be clinical stable in respect to pain treatment. We belive that the variability caused by serum concentration peaks is limited since samples obtained within a time interval of two hours after administration of a morphine rescue dose were excluded. However, it is important to recognize that in order to observe the exact pharmacological day-to-day variability of serum concentrations of morphine and morphine metabolites a design with a stable baseline morphine dose and a non-morphine alternative for breakthrough pain should be applied. Third, the use of rescue morphine implies that the daily morphine doses were not constant. However, the small changes in daily morphine doses can not explain the observed day-to-day variability.

In this study we assessed clinical symptoms related to opioid treatment in order to verify the stable intensities of symptoms during the study period. We did not attempt to explore the relationships between serum concentrations and clinical outcome measures. As a rule of thumb 25 patients are required per independent variable in order to give valid results in studies exploring the effects from factors predicting clinical observations [20]. Consequently, the size of this study was not sufficient to investigate the relationships between opioid serum concentrations and clinical symptoms.

## Conclusions

Morphine, M6G and M3G serum concentrations vary considerably in samples obtained on consecutive days. Such variability is present during stable morphine doses and stable clinical symptoms. The day-to-day variability was lower in patients receiving continuous sc morphine infusions compared with patients receiving oral morphine. These findings indicate that results from blood samples taken in order to assess a patient's pharmacological morphine status should be interpreted with the understanding of that variability is partly caused by day-to-day variation.

## Competing interests

The authors declare that they have no competing interests.

## Authors' contributions

PK, PH and JM participated in the design of the study, running of the study and preparation of the manuscript. SK, PCB and OD participated in the design of the study and preparation of the manuscript. KZ was responsible for measurements of morphine and morphine metabolite serum concentrations.

## Pre-publication history

The pre-publication history for this paper can be accessed here:


